# Ecological Thresholds of Toxicological Concern: A Review

**DOI:** 10.3389/ftox.2021.640183

**Published:** 2021-03-05

**Authors:** Mace G. Barron, Ryan R. Otter, Kristin A. Connors, Aude Kienzler, Michelle R. Embry

**Affiliations:** ^1^U.S. EPA, Office of Research & Development, Gulf Breeze, FL, United States; ^2^The Data Science Institute, Middle Tennessee State University, Murfreesboro, TN, United States; ^3^The Procter and Gamble Company, Cincinnati, OH, United States; ^4^European Commission, Joint Research Centre (JRC), Ispra, Italy; ^5^Health and Environmental Sciences Institute, Washington, DC, United States

**Keywords:** threshold of toxicological concern, mode of action, ecological, predicted no effect concentration, aquatic

## Abstract

The ecological threshold of toxicological concern (ecoTTC) is analogous to traditional human health-based TTCs but with derivation and application to ecological species. An ecoTTC is computed from the probability distribution of predicted no effect concentrations (PNECs) derived from either chronic or extrapolated acute toxicity data for toxicologically or chemically similar groups of chemicals. There has been increasing interest in using ecoTTCs in screening level environmental risk assessments and a computational platform has been developed for derivation with aquatic species toxicity data (https://envirotoxdatabase.org/). Current research and development areas include assessing mode of action-based chemical groupings, conservatism in estimated PNECs and ecoTTCs compared to existing regulatory values, and the influence of taxa (e.g., algae, invertebrates, and fish) composition in the distribution of PNEC values. The ecoTTC continues to develop as a valuable alternative strategy within the toolbox of traditional and new approach methods for ecological chemical assessment. This brief review article describes the ecoTTC concept and potential applications in ecological risk assessment, provides an overview of the ecoTTC workflow and how the values can be derived, and highlights recent developments and ongoing research. Future applications of ecoTTC concept in different disciplines are discussed along with opportunities for its use.

## TTC Foundation and History

The threshold of toxicological concern, or TTC concept, is an established risk assessment tool for determining a screening-level human exposure limit that poses negligible risk for groups of chemicals with limited data [reviewed in European Food Safety Authority and World Health Organization (2016)]. The TTC evolved following the US FDA's Threshold of Regulation, which was initially developed to assess human safety of indirect food-contact substances and was underpinned by carcinogenicity data from animal studies (USFDA, 1995). The approach was subsequently expanded into a tiered-TTC for oral exposures in which chemicals are assigned into different potency bins spanning over four orders of magnitude that cover both cancer and non-cancer endpoints (Kroes et al., 2004). The TTC concept applied in an ecological context (ecoTTC) establishes a concentration expected to have a *de minimis* probability of negative effects on aquatic communities for a group of toxicologically or chemically similar compounds (Belanger et al., 2015). The promise of the ecoTTC approach has been noted by the European Commission's EURL ECVAM Status Reports on Alternative Methods (Zuang et al., 2019), Environment and Climate Change Canada (ECCC, 2016; Health Canada and ECCC, 2018) and the Swedish Chemicals Agency (KEMI, 2020). Additionally, the U.S. Environmental Protection Agency has recognized the promise of the TTC approach generally within their prioritization strategy (USEPA, 2018).

EcoTTCs are computed from a percentile (e.g., 5%) of the statistical (probability) distributions of Predicted No-Observed Effect Concentrations (PNECs) for chemicals grouped by structural attribute (category), mode of action (MOA), or functional use. The concept of deriving threshold values to help aid ecological risk assessment (ERA) has been previously discussed by several researchers, each focusing on various distribution approaches and applications (De Wolf et al., 2005; Gross et al., 2010; Williams et al., 2011; Mons et al., 2013; Belanger et al., 2015; Gutsell et al., 2015; Wang et al., 2020). Although generally based on the human health TTC concept, application of this approach to the ecological space brings significant challenges. The human health TTC relies on chronic NOAEL values and extrapolations from sub-chronic studies, with the focus being on development of lifetime threshold values (Kroes et al., 2004). In the broadest sense, the goal of ERA is the protection of an ecological community or ecosystem, focusing on both acute and chronic effects. This broad scope presents a significant challenge when focusing on a specific aquatic habitat or environment with multiple trophic levels and taxa (fish, invertebrates, algae/plants, and amphibians), and when applied to protecting populations in diverse ecosystems (freshwater, marine, estuarine, etc.).

In addition to the broad coverage required in ERAs, there is increasing need to evaluate a large number of chemicals *via* international regulatory mandates, but with considerable resource limitations and a reduced reliance on vertebrate testing. Use of the ecoTTC provides for maximum use of existing knowledge. The approach allows for screening-level assessments of chemicals with little or no toxicity data and can provide “first-cut” guidance for product development. Conservative allowable exposure estimates for low-production volume chemicals can be derived and hazard assessments can be performed for groups of compounds for which QSARs are not available. As with various *in silico* or *in vitro* approaches, the ecoTTC is meant to be used as a screening level tool in a weight of evidence approach. The ecoTTC approach can be useful for assessing chemicals at early tiers of the risk assessment process by estimating hazard levels for chemicals that lack toxicity data or fall outside the domain of QSAR approaches, guiding product development discussions, or by assisting read-across or category justifications (Belanger et al., 2015). The ecoTTC approach also has the potential to reduce the need for vertebrate testing (e.g., fish) by developing effect concentrations for groups of chemicals with only limited chemical-specific *in vivo* test data.

Statistical approaches analogous to the ecoTTC have been applied in the ecotoxicity assessments of chemical groups including endocrine disrupting compounds (Gross et al., 2010) and diverse industrial compounds (Gutsell et al., 2015). These approaches have also been used in the development of MOA-based chemical toxicity distributions (CTDs) (Williams et al., 2011; Wang et al., 2020). An international collaboration under the Health and Environmental Sciences Institute (HESI) was established in 2014 to address challenges related to developing, applying, and implementing useful ecoTTC concepts and methods. Since its formation and publication of the paper outlining the concept (Belanger et al., 2015), this multi-stakeholder group has advanced the ecoTTC approach. Actions have included developing an open-access EnviroTox database and analysis tools (Connors et al., 2019), determining and exploring MOA schemes and assignments applicable to aquatic toxicity (Kienzler et al., 2017, 2019), assessing acute to chronic relationships in algal species, and applying the concept to a case study on chemical mixtures (Kienzler et al., 2020). The objectives of this article are to briefly describe the ecoTTC concept and potential applications in ERA, provide an overview of the ecoTTC workflow and how the values can be derived, and highlight recent developments and ongoing research. All acronyms are defined in Table 1. Finally, a vision for the future of the ecoTTC concept will be discussed regarding opportunities related to the approaches used for ecoTTC and how they may be applicable to different disciplines.

**Table 1:** Definitions of acronyms used in the review of the ecoTTC approach.

**Acronym**	**Definition**
AF	Application Factor
AOP	Adverse Outcome Pathway
ASTER	ASsessment Tools for the Evaluation of Risk
CAS	Chemical Abstract Service
CBR	Critical Body Residue
CTD	Chemical Toxicity Distribution
ECCC	Environment and Climate Change Canada
ECHA	European Chemicals Agency
ECOSAR	Ecological Structure Activity Relationships
ecoTTC	Ecological Threshold of Toxicological Concern
ERA	Ecological Risk Assessment
HC5	Hazardous Concentration for 5%
iTTC	Internal Threshold of Toxicological Concern
KEMI	Swedish Chemicals Agency—Kemikalieinspektionen
LCL	Lower Confidence Limit
MOA	Mode of action
NAM	New Approach Methodologies
NOAEL	No Observed Adverse Effect Level
OECD	Organization for Economic Cooperation and Development
PNEC	Predicted no effect concentration
QSAR	Quantitative Structure Activity Relationship
SIFT	Stepwise Information-Filtering Tool
SSD	Species sensitivity distribution
TTC	Threshold of toxicological concern
UCL	Upper Confidence Limit
USEPA	United States Environmental Protection Agency

## ECOTTC Conceptual Approach

Similar to the human health TTC, an ecoTTC can be used when little or no toxicity information are available. However, there are several important conceptual and practical differences between these tools. Unlike the human health TTC, which is focused on protecting one species (humans) using data from a few surrogate species (e.g., rat, rabbit, mouse), the ecoTTC must harmonize and interpret data across multiple species and trophic levels (e.g., algae, invertebrates, and fish). Because of this difference, both the mathematical approach and the interpretation of data must be handled differently.

**Figure 1:**
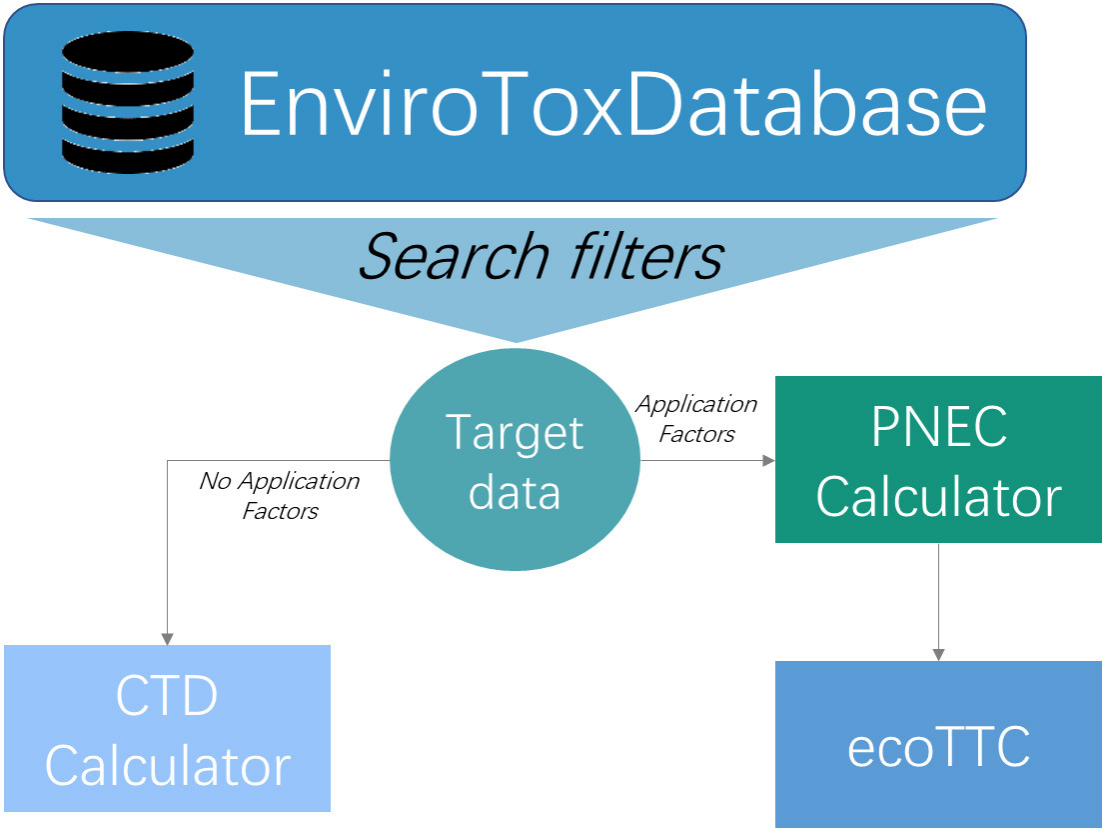
Schematic outlining the process for deriving a CTD (chemical toxicity distribution) or ecological threshold of toxicological concern (ecoTTC) using the EnviroTox platform (Connors et al., 2019). Targeted distributions can be created by using specific filtering criteria (e.g., MOA, chemical category, phys/chem property). The Predicted No Effect Concentration (PNEC) calculator is used to apply the correct, region-specific assessment factor based on the relative amount of data available for each test compound.

In this section we will outline the major steps involved in calculating an ecoTTC. Throughout this section we will utilize examples from the EnviroTox platform (www.envirotoxdatabase.org), an established database and tool set that was developed to make ecoTTC calculations accessible for aquatic ecotoxicologists, ecological risk assessors and managers (Figure 1). For a detailed description of the EnviroTox Platform please refer to Connors et al. (2019) and the EnviroTox User Guide (https://envirotoxdatabase.org/index.php/documentation).

### Data Sources and Curation

As with any data-driven approach, hazard estimates must be accurate and reproducible. Studies with regulatory relevant experimental durations and biological endpoints (e.g., mortality, growth, and reproduction) were included in the EnviroTox database. In total, approximately 220,000 data records, from at least 10 different sources including USEPA ECOTOX, ECHA, and the OECD QSAR Toolbox were identified as potentially useful. For the EnviroTox database, the Stepwise Information-Filtering Tool (SIFT) method (Beasley et al., 2015) was applied to select and curate aquatic toxicological data (Connors et al., 2019) such that the dataset was fit for purpose with regard to the ecoTTC application (e.g., data traditionally used for PNEC derivation). After applying SIFT criteria (e.g., data relevance, validity, and acceptability), less than 100,000 records were considered to be of high enough quality for inclusion.

Deliberate choices must be made during curation so uniform data types exist on all records. Specific examples include the definitions of acute and chronic that are specific to each species/trophic level, harmonization of chemical information [e.g., Chemical Abstract Service (CAS) registry number, chemical name, physico-chemical properties], and classification into MOA groups (e.g., Verhaar, OASIS, ASTER; Kienzler et al., 2019). Details of the specific curation criteria used for the development of the EnviroTox database and database coverage (species, chemicals, effects) are provided in Connors et al. (2019) and the user guide (Health and Environmental Sciences Institute, 2018).

### Selecting Data, Determining Application Factors, Calculating PNEC Values

An ecoTTC analysis can be made using different data subsets depending on the question being asked, including datasets specific to a certain MOA, or chemical category or functional use category. The ability to filter and subset the EnviroTox database to establish a data subset of interest was intentionally built into the ecoTTC analysis framework through the application of Boolean logic (Figure 1).

After the target dataset is selected, geometric means are generated separately for the acute and chronic hazard data for each chemical at the species level. If there is more than one species in a given trophic level (algae, invertebrate, and fish) for any chemical, then a trophic level geometric mean is calculated, resulting in a single value at each trophic level and data type (acute or chronic) for each chemical.

In order to convert these values to a chemical-specific PNEC, an application factor (AF) is applied to each chemical. An AF, also known as a safety or uncertainty factor, is used to account for the degree of conservatism or uncertainty applied to individual chemicals based on regulatory criteria or chemical screening protocol. Regional in nature, determining the appropriate AF depends on the amount and types of data available for each chemical in the target dataset, as well as the protection goal, and can be jurisdiction-specific. As previously shown in Hahn et al. (2014), inconsistent selection of AFs and the uneven handling of acute and chronic experimental data can result in PNECs varying more than three orders of magnitude. To reduce uncertainty and improve consistency, a codified logic was established to transparently select the AF for PNEC derivation.

**Figure 2:**
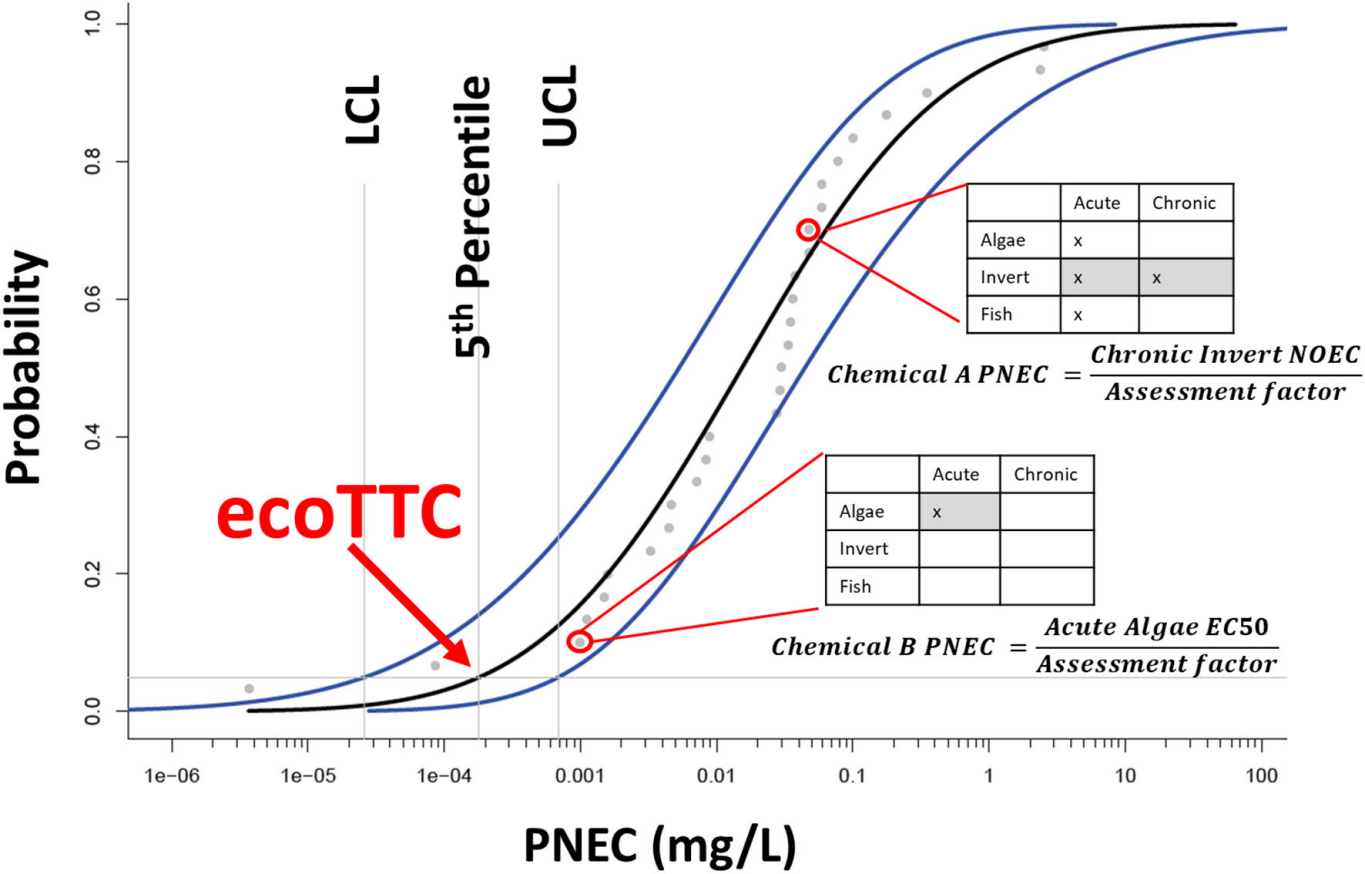
Example ecological threshold of toxicological concern (ecoTTC) probability distribution. Each data point in the distribution represents a chemical-specific predicted no effect concentration (PNEC) value. PNECs are calculated based on the relative amount of data (acute and chronic, for each trophic level). A shaded cell in the matrix tables indicates the trophic level with the lowest effect concentration; this value is divided by the appropriate assessment factor to derive the PNEC for each chemical in the distribution. In this example the ecoTTC is the 5th percentile of the distribution and is bounded by the lower (LCL) and upper (UCL) confidence limits.

The magnitude of the AF is selected by a predetermined combination of (1) the diversity of the data available (e.g., number of trophic levels represented by acute and chronic data) and (2) the region of interest (USA vs. Europe) (see EnviroTox database user guide for details). This value is applied to the most sensitive data group (e.g., chronic—invert; acute—algae) resulting in a single value for each chemical in the target database (Figure 2). This single value is a chemical-specific PNEC that is used to populate the ecoTTC probability distribution.

### ecoTTC Calculation and Interpretation

Once all chemical-specific PNECs have been determined in the target dataset, they are plotted using a cumulative frequency distribution curve (Figure 2). The fifth percentile PNEC value (PNEC_0.05_) is defined at the ecoTTC threshold value. Upper and lower confidence levels (UCL, LCL) reflecting the 95% confidence intervals can then be calculated.

## Toxicity Threshold Estimation With Envirotox

It is unlikely that a single, static threshold value will be derived for all chemicals. Instead, it is anticipated that unique ecoTTC thresholds may be created based on MOA, chemical class, regional practices or regulatory considerations, or other chemical grouping approaches. This remains an active area of investigation and opportunity for additional research. A relevant or fit for purpose model requires the selection of appropriate hazard data that meets a specific purpose or regulatory context. Before generating an ecoTTC, several considerations must be made. Which species or trophic level should be included in the distribution? Which biological endpoints should be considered? What applications factors should be applied, if any? What chemical domain would provide a meaningful output?

A high degree of flexibility was intentionally built into the EnviroTox database and the associated analysis tools to allow for data filtering and sub-categorization. The user can critically evaluate and establish a chemical and/or biological domain of applicability through the selection and application of relevant filters (Figure 1). Several physico-chemical properties are contained within the EnviroTox database including molecular weight, solubility, and logKow. Upper and/or lower bound filters can be set for these parameters. This allows the user to explicitly incorporate considerations of bioavailability or chemical domain of applicability. The EnviroTox database also contains Boolean descriptors to identify halogenated compounds and metals. In some cases, experimental conditions, such as water hardness, may significantly alter study interpretation and conclusions, especially for metals. In cases such as these, a careful review of the original citation would be needed before these experimental values could be used with confidence in most envisioned ecoTTC applications. Other filters for determining chemical groupings in EnviroTox include MOA and chemical class [e.g., ECOSAR class (USEPA, 2012) or US EPA New Chemical Categories (USEPA, 2010)].

Aquatic MOA can be classified using different approaches: using general structural rules, MOA-specific QSARs based on the presence of sub-structural fragment, expert judgement, or a mix of those. Each of these approaches may provide a different answer. A comparison of the main existing MOA classification frameworks for aquatic toxicity (Verhaar classification scheme, ASTER, and the U.S. EPA MOATox classification) was done (Kienzler et al., 2017) and found that only 42% of the chemicals classified within those three schemes were in agreement, whereas there was no agreement for 7% of the chemicals, and a partial agreement for the remaining chemicals. This was the basis for the development of a consensus MOA classification in the EnviroTox database (Kienzler et al., 2019). The consensus MOA classification is a binary classification which distinguishes narcotic chemicals from non-narcotic chemicals (i.e., which are expected to show a higher toxicity). This consensus classification is based on the individual assignments by Verhaar, ASTER, MOATox, and OASIS schemes. A confidence score for this new consensus classification is also derived based on the level of agreement between the four individual QSAR models.

When dealing with more than a single species, chemical classification can be complicated because of the uncertainly associated with assigning a single MOA across multiple compounds and taxonomic groupings. MOA designations have been largely created based on vertebrate data (e.g., Barron et al., 2015). Aquatic MOA classifications are mainly based on fish acute toxicity data, some even focusing on only a single species (e.g., Russom et al., 1997). Therefore, MOA classifications may not be applicable to all trophic levels due to the absence of a specific receptor or toxicity pathway (e.g., neurotoxicants MOA not relevant for algae; photosynthesis inhibitor not relevant for vertebrates). Because ecoTTCs are based on chemical-specific PNECs, the generation of a MOA-based ecoTTC may require careful review of the data available for each trophic level to ensure consistency in the chemical grouping applied. For example, Gutsell et al. (2015) and Wang et al. (2020) noted uncertainties with assigning MOA in developing ecoTTCs for diverse groupings of chemicals. EcoTTCs can also be derived using groupings based on chemical class, category or use. For example, Wang et al. (2020) noted that ecoTTC values for acetylcholinesterase inhibitor insecticides were lower for organophosphate compounds than for carbamates.

The choice of species or trophic level to include in the distribution should be carefully considered. The EnviroTox database is a collection of conventional, whole organism ecotoxicity studies measuring experimental endpoints with regulatory relevancy (i.e., mortality, growth, and reproduction). Therefore, the dataset is biased toward species tested under various regulatory programs. Currently >68% of the fish toxicity data are derived from 11 species and invertebrate data are dominated by only four freshwater species (Connors et al., 2019). High variability among reported toxicity values for single chemicals and species has been known for decades (Raimondo et al., 2009). Variation in toxicity values greater than 10-fold can result from differences in culture and test conditions, organism life stage, and analytical verification of exposure concentrations in different testing laboratories (Raimondo et al., 2009). In the EnviroTox database, geometric means were computed from the curated data to establish a single central tendency value for each species, endpoint, and chemical combination that were then used to derive PNECs. Geometric means based on limited data (e.g., three or less toxicity values; wide range in values) have greater uncertainty than those based on larger datasets.

Relative data availability per trophic level can significantly impact the resulting ecoTTC. PNECs by definition are inherently conservative as they predict a concentration where *no effect* will be observed. Large AFs are applied to data poor chemicals, reflecting the inherent uncertainty that comes with limited data. EcoTTC distributions created using all available data, including data poor chemicals (e.g., a single acute toxicity value with an AF of 1,000), have PNEC_0.05_ approximately 50% lower than distributions created from datasets that contain acute or chronic datasets for all three trophic levels (HESI, unpublished). Therefore, it is important to consider the relative amount of data, and the size of the applied assessment factor, for each compound in an ecoTTC distribution. Additional filters have been added to create custom ecoTTC distributions that allow inclusion or exclusion contain of compounds based on the magnitude of the assessment factor that was applied in generating the PNEC.

During the 2017 HESI international ecoTTC workshop (HESI, 2017) several participants noted concerns about the use of AFs (and by extension, PNECs) in ecoTTC distributions. There was concern that this may result in the derivation of an overly conservative *de minimis* threshold value. To meet this need, tools were developed to construct chemical toxicity distributions (CTDs) (Figure 1). ecoTTCs and CTDs are conceptually very similar. EcoTTCs are distributions of PNECs, leveraging an existing regulatory framework for collapsing acute and chronic data from multiple trophic levels into a single value per chemical. By contrast, CTDs are probability-based statistical distributions of experimental effect concentrations (Table 2). Stepping away from the PNEC framework and AFs allows more biological freedom when creating CTDs.

**Table 2:** Comparison of three probability-based statistical distributions of toxicity values to human health thresholds of toxicological concern (TTC): ecological threshold of toxicological concern (ecoTTC), chemical toxicity distribution (CTD), and species sensitivity distribution (SSD).

**Parameter**	**ecoTTC**	**CTD**	**SSD**	**Human health TTC**
Species	All (same exposure medium)	All (same exposure medium)	All (same exposure medium)	Standard mammalian test species
Trophic levels	Fish, invert, and algae	Selected by user (e.g., all trophic levels, fish only)	All available	One
Test duration/type	Acute *and* chronic data, combined	Acute, chronic *or* chronic with extrapolated acute values	Acute *or* chronic, depending on application	Various
Dataset	Chemical PNEC values	Effect concentration for a given chemical	Effect concentration observed for a given species	Effect concentration
Number of chemicals in distribution	Multiple	Multiple	One	Multiple
Application/uncertainty factors	Chemical-specific^a^	Not directly applied	Not directly applied	Various^b^

a *Applied to each chemical based on extrapolation uncertainty or regulatory jurisdiction*.

b *Applied to individual no effect values to account for inter- and intra-species variability*.

CTDs can be created using a single species, a single trophic level, or by combining all data together. Conceptually, the human health TTCs that are developed by combining several mammalian species (e.g., rat, mouse, and rabbit) are analogous to a trophic level ecological CTD. When one trophic level is uniquely sensitive to a class of compounds (e.g., fish to neurotoxicants, algae to photosynthesis inhibitors), a species-specific or trophic-level-specific CTD may be more insightful. EcoTTCs rely on PNEC logic for combining acute and chronic data. Without this framework for collapsing data, CTDs can be built on acute *or* chronic data. Alternatively, chronic data could be supplemented with additional chemicals by estimating a chronic value from acute data using acute to chronic ratio extrapolations (Raimondo et al., 2009; May et al., 2016).

The species sensitivity distribution (SSD) is a routinely used environmental hazard assessment tool (Newman et al., 2000). SSDs are probability distributions of toxicity values that are mathematically and conceptually similar to a CTD. CTDs are distributions of *multiple* chemicals, whereas SSDs are generated using toxicity data for a single compound. Low AFs (1–5) may be applied to SSD HC_5_ values in order to derive a chemical-specific PNEC value. There are future plans for incorporating SSD-derived PNECs in ecoTTC distributions.

Although the use of the geometric mean allows the user to give less weight to extreme data in the dataset for one species, the ecoTTC remains a conservative approach because of the conservatism of two components of the derivation: the chemical-specific PNEC calculation and the use of a 5th percentile of the probability distribution. PNEC conservatism is driven by the most sensitive species in the dataset and an AF (5–1,000) to ensure protection of potentially more sensitive species without experimental data. The use of the threshold value, set as the 5th percentile of the whole distribution, ensures that the ecoTTC encompasses 95% of compounds in the chemical group. In contrast, the CTD does not include the AF in PNEC derivation, which means that it is expected to be an order of magnitude or more above the ecoTTC value (Kienzler et al., 2019) and gives a value which is meant to cover 95% of the chemicals/species combination considered in the distribution. An alternative approach can be to derive a CTD and then apply an AF to the 5th percentile to cover the “remaining” chemical/species combinations that are not included in the distribution, as it is done in the SSD approach. In this case the assessment factor usually goes from two to five to cover the “missing” species in the distribution.

## Future Developments and Research Needs

As discussed above, the database is biased toward laboratory species tested in a regulatory context. Additional investigation of the dataset would be interesting to better understand how the toxicity data of the non-standard test organisms are situated in the distribution. Are they more frequently identified as outliers in the distribution, and are those species covered by the threshold derived, or show higher sensitivity than laboratory species?

Current well-established human health TTC values are focused on exposures that are assessed as external oral ingestion, whereas internal exposures based on blood concentrations can be more relevant for other exposure routes, such as inhalation (Tluczkiewicz et al., 2016) or for substances with low oral absorption. Additionally, recent efforts in human health TTCs have focused on deriving an internal threshold of toxicological concern (iTTC) that may ultimately enable route-to-route extrapolation and TTC development for multi-route exposures (Ellison et al., 2019; Blackburn et al., 2020). Because aquatic organism exposures mainly occur through water, there is less need to address multi-exposure routes. However, the development of an internal ecoTTC for aquatic organisms could address variation in interspecies bioavailability and allow better integration with *in vitro* toxicity data. A similar approach to the iTTC has been developed in ecotoxicology, the critical body residue (CBR) approach (McElroy et al., 2011). In the CBR approach, specific tissue concentrations of a chemical in an organism are associated with adverse biological effects. When the internal CBR concentration is reached, the adverse effect will be triggered, regardless of external exposure conditions (McCarty and Mackay, 1993). Therefore, a CBR can be an ideal metric of the intrinsic toxicity of a chemical based on concentrations in the organism rather than relying on the external exposure concentration in water.

Additional research and development are needed to further investigate the potential use of ecoTTC and CTD threshold values in assessment of emerging chemicals and chemical mixtures. Cronin (2017) suggested ecoTTCs could facilitate the assessment of the chronic toxicity of mixtures by focusing AOP and QSAR development on those compounds that exceed threshold values. Recent research on ecoTTCs and CTDs in a mixture assessment determined that the derived values were adequately conservative, but application to all chemicals in a mixture was limited because of compounding conservatism (Kienzler et al., 2020). Ongoing research compares threshold values to current water quality criteria from different geographic and regulatory jurisdictions to better understand if ecoTTC or CTD values could be applied to the development of water quality standards for chemicals with limited toxicity data. The TTC approach has been applied to endocrine disruptors although the broad applicability of the approach is uncertain (Gross et al., 2010). For example, some authors have concluded that TTC should not be used for endocrine disrupting chemicals because of the uncertainty in low-dose extrapolation and the complexity of effects (Kroes et al., 2004). Also, some endocrine effects may not be detectable in standardized tests with conventional test organisms, though progress has been made in the past 10 years with the development of several new test guidelines focusing on endocrine disruption. Future research could explore chemical grouping options for endocrine active compounds within the EnviroTox database, with a focus experiments containing endocrine related endpoints and trophic level sensitivity.

In the process of developing a human health TTC, some compound classes were excluded because of lack of representation in the supporting database, such as proteins, metals, or metal-containing compounds (Patlewicz et al., 2018), lack of inclusion of the endpoint (e.g., protein allergenicity), evidence of bioaccumulation, or high-potency carcinogens (Nelms et al., 2019). Additional work is still needed to define what chemicals may similarly be out of scope of an ecoTTC distribution, but it should be noted that none of the above chemicals were categorically excluded in developing the EnviroTox database, though metals have been flagged.

## Conclusions

EcoTTCs have seen significant advancement since the review of Belanger et al. (2015), including increasing evaluation for use in environmental risk assessment and development of a public domain computational platform for PNEC and ecoTTC derivation with aquatic species toxicity data (https://envirotoxdatabase.org/). Ongoing research and future development needs include assessing MOA-based and compound-based chemical groupings, optimizing logic flows in PNEC derivation, and determining the influence of taxa composition in the distribution of PNECs and magnitude of ecoTTC values. Uncertainty in estimated PNECs and conservatism in ecoTTCs may be quantified through comparison of CTDs and ecoTTCs to existing regulatory values and international screening benchmarks.

Human health-based TTCs have a long history of use as a screening method of determining acceptable levels of chemical exposures in food products and more recent applications in global chemical regulation [e.g., USEPA, 2018]. ecoTTCs expand the application of the TTC framework to the diversity of ecological species through a practicable approach for assessing groups of data poor chemicals that may pose risks to ecological receptors. The recent development of the EnviroTox platform provides a web-based public domain computational tool for transparent and reproducible ecoTTC estimates based on customized user defined inputs. Use and application of ecoTTCs for regulatory purposes, similar to application of the human health approach, has already been highlighted within several agency documents and strategies (ECCC, 2016; Health Canada and ECCC, 2018; Zuang et al., 2019; KEMI, 2020). The approach is seen as a valuable alternative strategy within the toolbox of traditional and new approach methods (NAMs) for ecological chemical assessment. Additional work on illustrative and real-world examples will aid in the use and uptake of this important approach.

## Author Contributions

All authors listed have made a substantial, direct and intellectual contribution to the work, and approved it for publication.

## Conflict of Interest

The authors declare that the research was conducted in the absence of any commercial or financial relationships that could be construed as a potential conflict of interest.
